# Active Actuating of a Simply Supported Beam with the Flexoelectric Effect

**DOI:** 10.3390/ma13071735

**Published:** 2020-04-08

**Authors:** Mu Fan, Hequn Min

**Affiliations:** 1 State Key Laboratory of Mechanics and Control of Mechanical Structures, Nanjing University of Aeronautics and Astronautics, Nanjing 210010, China; 2 Key Laboratory of Urban and Architectural Heritage Conservation, Ministry of Education, School of Architecture, Southeast University, Nanjing 210096, China; hqmin@seu.edu.cn

**Keywords:** flexoelectric effect, simply supported beam, active actuating, size effect

## Abstract

Piezoelectric materials with the electro-mechanical coupling effect have been widely utilized in sensors, dampers, actuators, and so on. Engineering structures with piezoelectric actuators and sensors have provided great improvement in terms of vibration and noise reduction. The flexoelectric effect—which describes the coupling effect between the polarization gradient and strain, and between the strain gradient and electric polarization in solids—has a fourth-rank order tensor electro-mechanical coupling coefficient, and in principle makes the flexoelectricity existing in all insulating materials and promises an even wider application potential in vibration and noise control. In the presented work, a flexoelectric actuator was designed to actuate a simply supported beam. The electric field gradient was generated by an atomic force microscopy probe. Flexoelectric control force and moment components could be induced within the flexoelectric control layer. As flexoelectricity is size-dependent, the key parameters that could affect the actuating effect were examined in case studies. Analytical results showed that the induced flexoelectric control moment was strongly concentrated at the probe location. The controllable transverse displacement of the simply supported beam was calculated with the modal expansion method. It was found that the controllable transverse displacement was dependent on the probe location as well.

## 1. Introduction

In the past few decades, devices using piezoelectricity, e.g., actuators and transducers, have been invented and applied to various engineering systems and applications. Piezoelectricity is an electromechanical phenomenon which couples with the elastic and electric fields. In general, a piezoelectric material responds to mechanical forces and generates an electric charge/voltage or responds to an electric input and induces mechanical stresses or strains, which are known as the direct and converse piezoelectric effects, respectively. With the direct piezoelectric effect, Dietl et al. [1] proposed a Timoshenko model of transverse piezoelectric beam vibration and examined the frequency response of vibration-based energy harvesters. Zhang et al. [2] developed a generic linear and nonlinear piezoelectric shell energy harvesting theory based on a double-curvature shell. Due to its low cost, compact sensor size, and simple signal conditioning, piezoelectric sensing has also been applied in high-temperature applications, including accelerometers, surface acoustic wave sensors, ultrasound transducers, acoustic emission sensors, gas sensors, and pressure sensors for temperatures up to 1250 °C [3]. As the direct piezoelectric effect is usually utilized in engineering sensing and energy harvesters, the converse piezoelectric effect can be applied to structure control and active actuating. Kenan and Ismail studied the optimal piezoelectric vibration control of a Timoshenko beam [4] and Mindlin-type beam [5]. Shen et al. [6] adopted piezoelectric elements to control the vibration of an autobody thin-wall structure by simplifying the thin-wall structure to a square plate with a peripheral clamped boundary. Tzou [7] introduced the application of piezoelectric effects to various shell structures and proposed control strategies. With the piezoelectric sensing and actuating capability, Akira et al. [8,9] actively controlled sound transmission through a rectangular panel. In their study, piezoelectric sensors were distributed to specific locations to detect the system vibration signal and reduce the observation spillover. All these research works have proved that the piezoelectric effect can be effectively used in engineering sensing, vibration, and noise control.

Flexoelectricity describes the linear coupling between the electric field gradient and stress/strain or the electric polarization and strain gradient, which can be used as a complement to piezoelectricity. Compared to piezoelectricity, the flexoelectric coefficient is a fourth-rank order tensor and hence in principle exists in all insulating materials, while piezoelectricity is limited to non-centrosymmetric materials [10]. As a gradient coupling effect, the flexoelectric effect is size-dependent—it becomes increasingly prominent as the system size diminishes. Qi et al. [11,12] fabricated a stretchable energy harvester by coupling wavy piezoelectric nanoribbons with a bendable rubber substrate. The buckled PZT (or lead zirconate titanate, Pb[Zr(x)Ti(1 − x)]O_3_) nanoribbons can sustain large strain gradients and give a rise of up to 70% enhancement of the apparent piezoelectric performance as a result of the flexoelectric effect. By studying the electromechanical behavior of graphene-based nanocomposite beams, Kishor and Kundalwal [13] found that the flexoelectric and surface effects on the static response of GNC (graphene-based nanocomposite) nanobeams were significant and could not be ignored. Qi et al. [14] established a flexoelectric curved microbeam model based on the flexoelectric theory, incorporating strain gradient and polarization gradient. Both static bending and dynamic vibration were studied. The flexoelectric effect manifests itself when the intrinsic flexoelectric coupling is high. Electric field/polarization gradients may arise from non-uniformity of the polarization or electric field. Zhang et al. employed an atomic force microscope probe to generate an inhomogeneous electric field and successfully controlled the dynamic vibration of a cantilever beam with a flexoelectric actuator [15]. Fan et al. [16,17] studied the active actuating and vibration control of beams and plates with multiple flexoelectric actuators. Recently, a review work on flexoelectricity in solids showed that the flexoelectric effect, from its fundamental theory to its applications, is attracting more and more research attention [10].

In the current study, the converse flexoelectric effect was proposed to actuate a simply supported beam. The electric field gradient was generated by an atomic force microscopy (AFM) probe. The purpose of this study was to examine the key parameters that may influence the electric field gradient and validate the effectiveness of flexoelectric actuators in structure actuating. The distributions of the electric field gradient, flexoelectric control moment, flexoelectric modal force, and beam transverse displacement are discussed with respect to varying the flexoelectric layer thickness and AFM probe radius.

## 2. Materials and Methods

**Figure 1:**
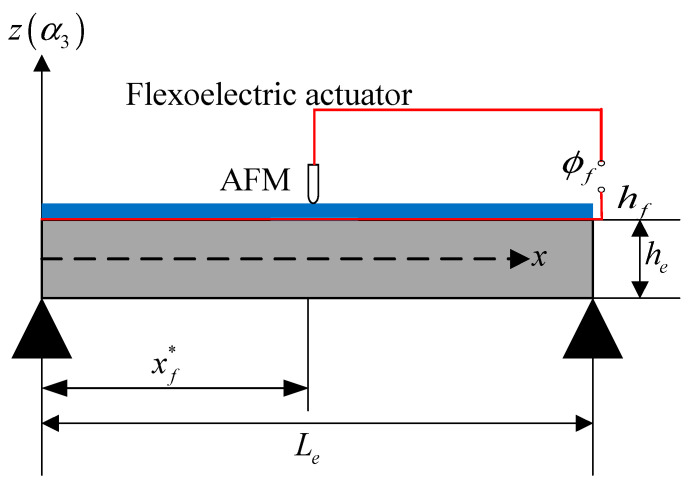
A simply supported beam with flexoelectric actuating.

The active damping technique is usually used in engineering vibration and noise control, and the simplest active damping is to bond a piezoelectric actuating layer to a fundamental structure so that the dynamic vibration can be controlled with the specific electric input. In this study, a simply supported beam was selected as the fundamental structure. On the top surface of the beam, a flexoelectric layer was perfectly bonded, serving as a control layer as shown in Figure 1. An AFM probe was applied to the flexoelectric layer, along with an electrode layer on the bottom of the flexoelectric layer but on the top of the elastic beam. When a certain electric input was applied to the bottom electrode layer and the AFM probe, an inhomogeneous electric field could be generated in the flexoelectric layer and the flexoelectric control force and control moment could be obtained relative to the converse flexoelectric effect. As mentioned previously, the flexoelectric effect relies on the gradient effect. In the current model, the electric field gradient was strongly affected by the probe radius and flexoelectric thickness. However, the overall actuating effect of the simply supported beam was also influenced by the beam parameters and boundary conditions as well.

As illustrated in Figure 1, a flexoelectric patch (*L_f_* for length, *h_f_* for thickness, and *b* for width) was perfectly bonded to the upper surface of the beam as a constraint surface. The electric field gradient was generated by an AFM (atomic force microscopy) probe (radius, *R*) and the location of the probe is denoted by x*_f_. The elastic beam parameters were as follows: elastic beam length *L_e_*, thickness *h_e_* and width *b*. The radius of the AFM probe and the layer thicknesses follow that were *R* << *h_f_* << *h_e_*, so that the stiffness effect on the dynamic model could be neglected and the beam structure could be modelled with a pure elastic beam model. The electric input to the AFM probe is denoted by *Φ_f_*.

### 2.1. The Electric Field

The electric field in this study was generated by the AFM probe on the top and the electrode layer on the bottom. Generally, the electric input is assumed to be a harmonic excitation with frequency *ω_f_*, amplitude $\phi_{f}^{a}$, and phase angle $\varphi_{f}$, as given in Equation (1):

(1) $$\phi_{f}=\phi_{f}^{a}e^{j\left(\omega_{f}t-\varphi_{f}\right)}$$

The electric field induced by the AFM probe under the current input is estimated as [18,19]:

(2) $$E_{3f}\left(x,z\right)=-\frac{\phi_{f}^{a}R\left(R+h_{e}/2+h_{f}-z\right)}{{\left[{\left(x-x_{f}^{*}\right)}^{2}+{\left(R+h_{e}/2+h_{f}-z\right)}^{2}\right]}^{\frac{3}{2}}}$$

Here, *E*_3*f*_(*x*, *z*) is the electric field in the transverse direction. The electric field gradient can be derived as:

(3) $$\frac{\partial E_{3f}}{\partial z}\;=-\frac{2R\phi_{f}^{a}}{{\left[{\left(x-x_{f}^{*}\right)}^{2}+{\left(R+h_{f}+h_{e}/2-z\right)}^{2}\right]}^{\frac{3}{2}}}+\frac{3R{\left(x-x_{f}^{*}\right)}^{2}\phi_{f}^{a}}{{\left[{\left(x-x_{f}^{*}\right)}^{2}+{\left(R+h_{f}+h_{e}/2-z\right)}^{2}\right]}^{\frac{5}{2}}}$$

From Equation (2), it can be found that along x direction, the electric field reaches the maximum value when $x=x_{f}^{*}$, right under the location of the probe. Along the transverse direction, it is a function of z and is inhomogeneous. The flexoelectric control stress $T_{xx}^{f}\left(x,z\right)$ can be written in terms of the electric field gradient and the flexoelectric coefficient as given in Equation (4):

(4) $$T_{xx}^{f}\left(x,z\right)=\pi_{12}\frac{\partial E_{3f}}{\partial z}$$

Here, $T_{xx}^{f}$ denotes the longitudinal normal stress induced by the flexoelectric patch and $\pi_{12}$ is the flexoelectric constant and will be given in the case studies.

With the stress component obtained in Equation (4), the control moment which can actuate the simply supported beam can be derived as [20]:

(5) $$\begin{array}{l} M_{xx}^{f}\left(x\right)=\frac{h_{e}+h_{f}}{2}\times \int_{h_{e}/2}^{h_{e}/2+h_{f}}T_{xx}^{f}\left(x,z\right)dz\\ =\frac{\pi_{12}R\left(h_{e}+h_{f}\right)\phi_{f}^{a}}{2}\left\{\frac{\left(R+h_{f}\right)}{{\left[{\left(x-x_{f}^{*}\right)}^{2}+{\left(R+h_{f}\right)}^{2}\right]}^{\frac{3}{2}}}-\frac{R}{{\left[{\left(x-x_{f}^{*}\right)}^{2}+R^{2}\right]}^{\frac{3}{2}}}\right\} \end{array}$$

In Equation (5), $M_{xx}^{f}$ denotes the flexoelectric moment induced by the flexoelectric patch. Parameters that can affect the control moment include the voltage amplitude $\phi_{f}^{a}$, structure thicknesses $h_{e}$ and $h_{f}$, and AFM probe radius as well as the probe location on the elastic beam $x_{f}^{*}$. Again, $h_{f}$ and R determine the size of the flexoelectric layer, which indicates that flexoelectric actuating is strongly size-dependent.

### 2.2. Dynamic Response of the Simply Supported Beam

The dynamic response of the simply supported beam under actuating can be described in terms of transverse displacement (i.e., $u_{3k}^{}\left(x,t\right)$) with the modal expansion method [21]

(6) $$u_{3k}^{}\left(x,t\right)=\sum_{k=1}^{\infty }\eta_{k}^{}\left(t\right)U_{3k}^{}\left(x\right)$$

where k denotes the vibration mode, $\eta_{k}$ denotes the $k^{th}$ modal participation factor (or the modal coordinate), and $U_{3k}$ is the mode shape function of the beam model, which can be expressed as in Equation (7) for the current of the simply supported beam [22]:

(7) $$U_{3k}^{}\left(x\right)=C_{k}\sin\lambda_{k}x$$

Here, $C_{k}$ is the modal amplitude of the $k^{th}$ mode; $\lambda_{k}L$ is the root of the characteristic equation; and the first three roots are $\lambda_{k}L=\pi ,\;$ 2π,  3π,(k=1,2,3). The equation of the mode participation factor (or the $k^{th}$ modal equation) can be written as:

(8) $${\ddot{\eta }}_{k}+2\zeta_{k}\omega_{k}{\dot{\eta }}_{k}+\omega_{k}^{2}\eta_{k}={\hat{F}}_{k}\left(t\right)$$

where $\omega_{k}$ is the $k^{th}$ natural frequency and $\zeta_{k}$ is the modal damping ratio, which can be defined as $\zeta_{k}=c/\left(2\rho h\omega_{k}\right)$, depending on the equivalent damping constant c. ${\hat{F}}_{k}\left(t\right)$ is the flexoelectric modal force, which is defined as:

(9) $${\hat{F}}_{k}^{}=\frac{1}{\rho hN_{k}}\int_{0}^{L_{e}}\left(\frac{\partial^{2}M_{xx}^{f}}{\partial x^{2}}\right)U_{3k}\left(x\right)dx$$

The controllable beam transverse displacement (i.e., $u_{3}^{}(x)$) can be expressed based on the modal force as:

(10) $$\begin{array}{ll} u_{3}^{}(x) & =\sum_{k=1}^{\infty }\frac{\;U_{3k}^{}(x){\hat{F}}_{k}^{}\left(x_{i}^{*}\right)e^{j(\omega t-\varphi_{}^{*})}}{\omega_{k}^{2}\sqrt{{\left(1-\frac{\omega^{2}}{\omega_{k}^{2}}\right)}^{2}+4\zeta_{k}^{2}{\left(\frac{\omega }{\omega_{k}^{}}\right)}^{2}}}\\ & =\sum_{k=1}^{\infty }\frac{\;U_{3k}^{}(x)\int_{0}^{L_{e}}\left(\frac{\partial^{2}M_{xx}^{f}\left(x-x_{f}^{*}\right)}{\partial x^{2}}\right)U_{3k}\left(x\right)dxe^{j(\omega t-\varphi_{}^{*})}}{\rho hN_{k}\omega_{k}^{2}\sqrt{{\left(1-\frac{\omega^{2}}{\omega_{k}^{2}}\right)}^{2}+4\zeta_{k}^{2}{\left(\frac{\omega }{\omega_{k}^{}}\right)}^{2}}} \end{array}$$

## 3. Results and Discussion

In the current study, the effect of the size of the flexoelectric patch on the dynamic response of the simply supported beam was studied. Both the flexoelectric patches were made of PVDF (Polyvinylidene Fluoride) and the elastic beam was made of polypropylene. The parameters are given in Table 1.

### 3.1. The Electric Field Gradient

Based on the converse flexoelectric effect, the induced flexoelectric stress was proportional to the electric field gradient. When the flexoelectric material was fixed, increasing the electric field gradient could be one possible way to enhance the flexoelectric effect. As mentioned before, in the current physical model the AFM probe radius and the flexoelectric patch thickness could affect the electric field gradient.

**Figure 2:**
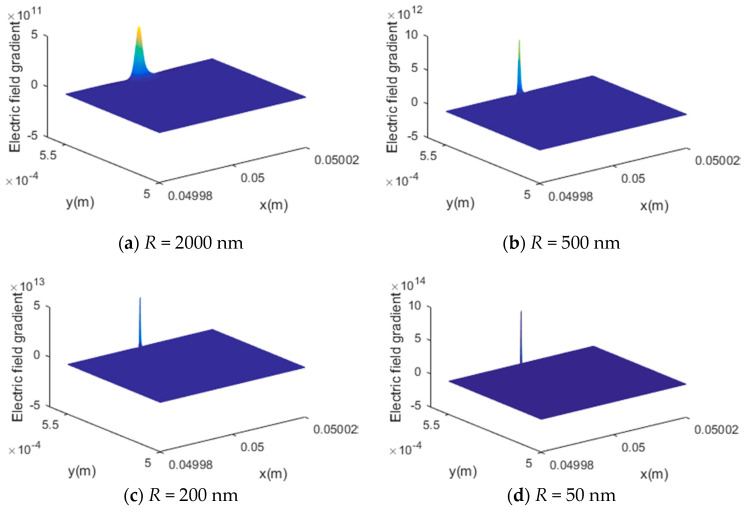
The effect of the AFM probe radius on the electric field gradient, (**a**) R = 2000 nm, (**b**) R = 500 nm, (**c**) R = 200 nm, (**d**) R = 50 nm.

**Figure 3:**
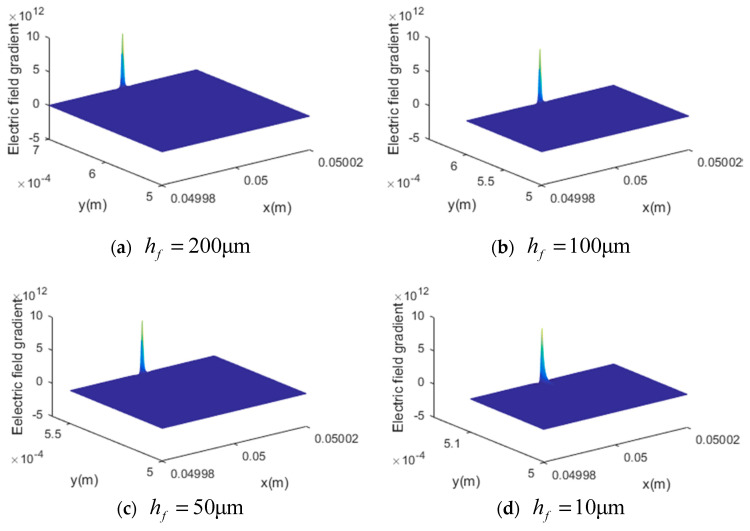
The effect of the flexoelectric layer thickness on the electric field gradient, (**a**) $h_{f}=200\;\mu m$, (**b**) $h_{f}=100\;\mu m$, (**c**) $h_{f}=50\;\mu m$, (**d**) $h_{f}=10\;\mu m$.

In Figure 2, when decreasing the AFM probe from 2000 nm to 50 nm, the maximum value of the electric field gradient increased significantly. Additionally, the distributions of the gradient along both the x axis and z axis were changed with different probe R radii. With a smaller probe radius, the electric field gradient became more concentrated. However, when decreasing the flexoelectric layer thickness, the maximum value of the gradient had barely no change, as shown in Figure 3. This can be explained by the electric field gradient expression given in Equation (3), in which the maximum value is obtained at $x=x_{f}^{*}$ and $z=h_{e}/2+h_{f}$, which makes it independent of $h_{f}$.

**Figure 4:**
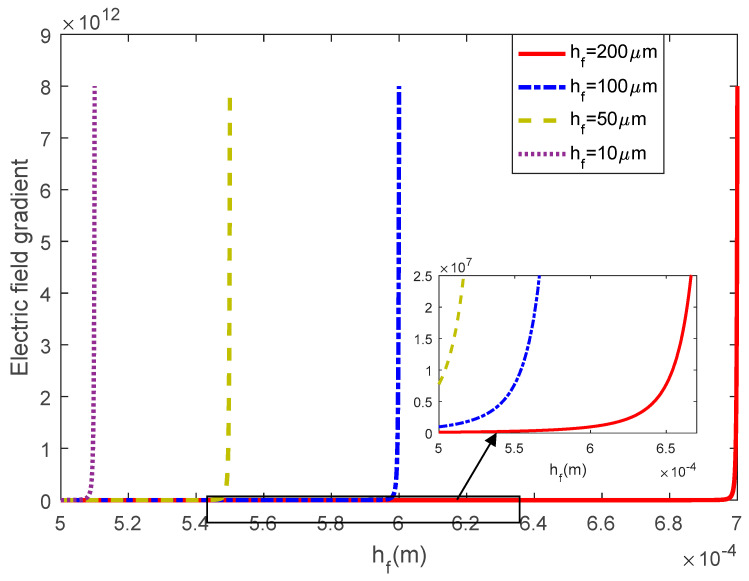
The electric field gradient when $x=x_{f}^{*}$ with different flexoelectric layer thicknesses.

However, by decreasing the flexoelectric layer thickness while the external electric input was fixed, the electric field distribution could be affected physically. To further study the influence of the flexoelectric layer thickness, the electric field gradient when $x=x_{f}^{*}$ is plotted in Figure 4. It was found that the electric field gradient near $\left(x_{f}^{*},h_{e}/2+h_{f}\right)$ increased sharply to the maximum value and was independent of the thickness $h_{f}$. However, as shown in the embedded figure in Figure 4, when it moved to $h_{e}/2$ along the z axis, the values of different layer thicknesses were differentiated. This meant that with a thinner flexoelectric layer, the minimum electric field gradient increased. However, since the maximum electric field gradient of each thickness was the same and always much larger than the minimum gradient value, the influence of the flexoelectric layer thickness on the flexoelectric effect could be limited.

### 3.2. The Flexoelectric Control Moment

**Figure 5:**
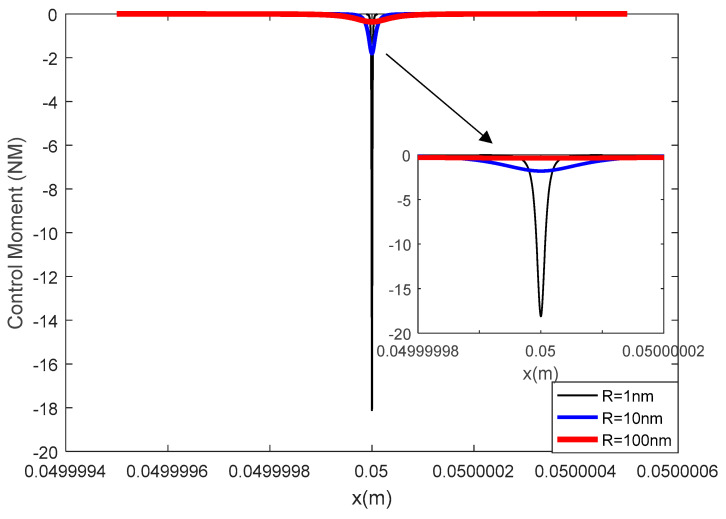
The effect of the AFM probe radius on the flexoelectric control moment.

**Figure 6:**
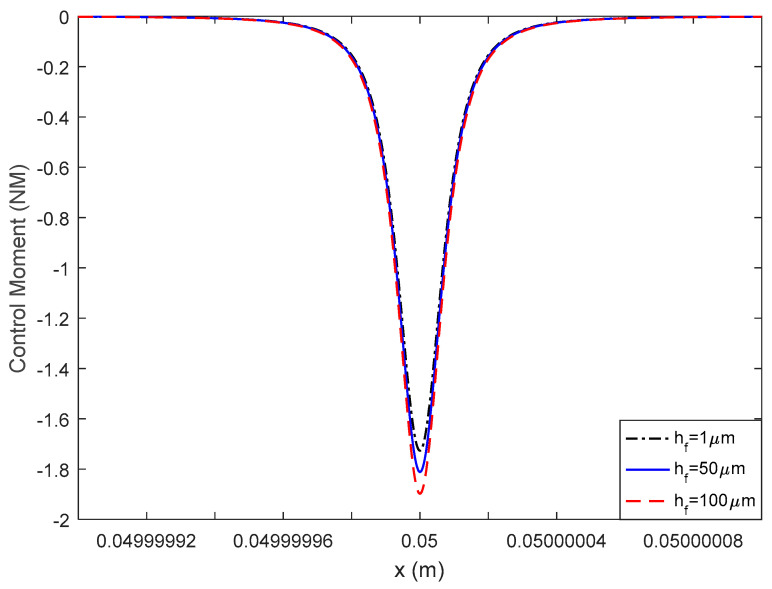
The effect of the flexoelectric patch thickness on the control moment.

A flexoelectric control force and control moment can be induced with an inhomogeneous electric field in the flexoelectric layer and thus actuate the elastic beam structure. For the beam mode used in this study, it was the moment components causing the vibration. In Figure 5 and Figure 6, the flexoelectric control moment distribution is illustrated versus the various AFM probe radii and flexoelectric patch thicknesses.

In Figure 5, it was found that the maximum value of the control moment increased sharply when decreasing the probe radius; in Figure 6, when increasing the flexoelectric patch thickness, the control moment increased. Generally, when decreasing the probe radius, the electric field gradient increased quickly, which resulted in an increase in the control moment. However, the effect of the thickness on the flexoelectric control moment could be complex. On the one hand, with a thinner flexoelectric layer, the electric field gradient could increase (minimum values increased while mixed values stayed the same as in the previous section), which was positive to the flexoelectric control moment. On the other hand, with a thinner flexoelectric layer, the moment arm decreased, which was negative to the flexoelectric control moment. The final effect of the flexoelectric layer thickness on the control moment was a balance of the electric field gradient and moment arm. In the current case, the moment arm change was more dominating and an increased control moment was observed with greater flexoelectric layer thicknesses.

Mathematically, there are two parts in the braces in Equation (5). The first part is dependent on both the probe radius and patch thickness, and the second part is dependent on the probe radius only. As the probe radius is much smaller than the patch thickness, the second part always dominates the total value of the control moment expression. With a decrease in R, the second part increases sharply, and with a decrease in R or in thickness, the first part decreases slowly. Hence, the flexoelectric control moment can be increased with a smaller probe radius and larger layer thickness, which is consistent with the physical explanation. However, it should be noted that the conclusion was made based on the assumption that $R<<h_{f}<<h_{e}$. Otherwise, the beam should be modelled as a laminated beam instead.

### 3.3. The Flexoelectric Modal Force

With the modal expansion method, the controllable transverse displacement of the simply supported beam can be written in terms of the modal participation factor and mode shape function. The flexoelectric modal force, which determines the modal participation factor, is examined in this section with regard to the AFM probe radius and flexoelectric patch thickness.

**Figure 7:**
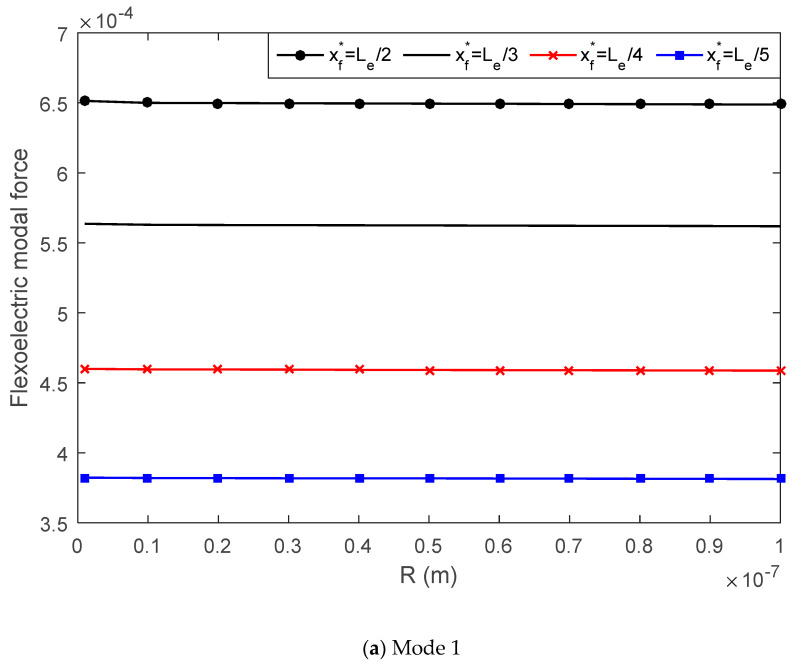

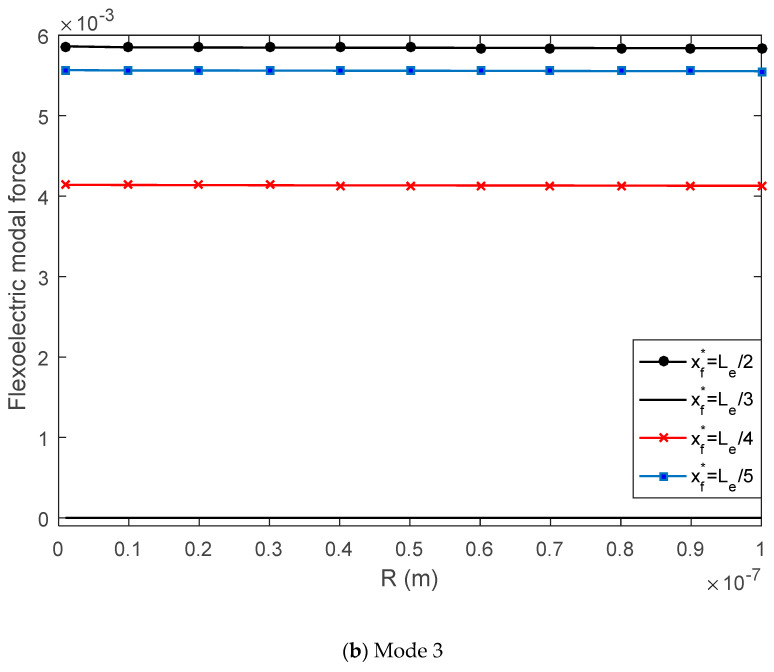
The effect of the probe radius on the flexoelectric modal force, (**a**) mode 1, (**b**) mode 3.

**Figure 8:**
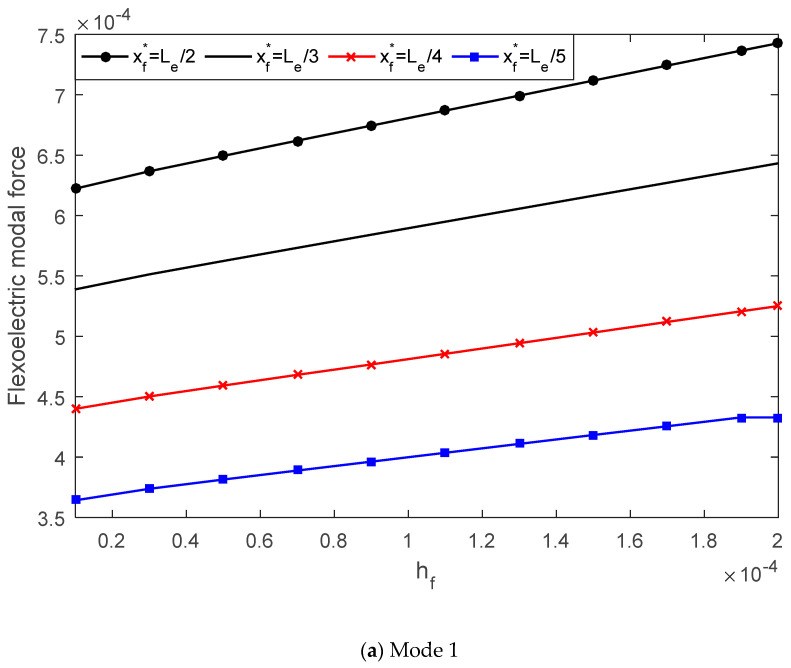

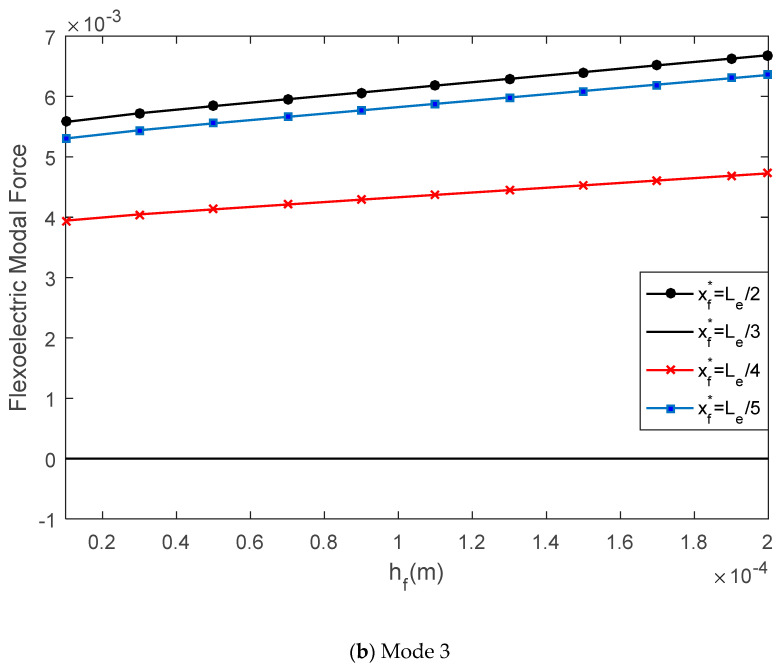
The effect of the flexoelectric patch thickness on the flexoelectric modal force, (**a**) mode 1, (**b**) mode 3.

In Figure 7, the mode 1 and 3 modal force is plotted versus the probe radius. It was found that when increasing the probe radius from 10 nm to 100 nm, the flexoelectric modal force decreased in both the mode 1 and mode 3 cases. In Figure 8, when increasing the flexoelectric patch thickness, the modal force of both mode 1 and mode 3 increased. This proves that the influence of the patch thickness on the flexoelectric effect is a balance of the electric field gradient and moment arm. Furthermore, when varying the location of the AFM probe on the elastic beam from $x_{f}^{*}=L_{e}/2$ to $L_{e}/5$, the flexoelectric modal force changed significantly. In the case of mode 1, moving the probe from the middle of the beam ($x_{f}^{*}=L_{e}/2$) to the end ($x_{f}^{*}=L_{e}/5$) caused the modal force to decrease consequently. However, in the case of mode 3, when $x_{f}^{*}=L_{e}/3$ the flexoelectric modal force was zero. The influence of the AFM probe location on the flexoelectric modal force was induced by the modal shape functions. The flexoelectric modal force was affected by both the flexoelectric control moment and beam vibration mode shape. Hence, it is possible to enhance the flexoelectric actuating effect by increasing the electric field gradient and adjusting the actuator location to their optimal mode-dependent locations.

### 3.4. The Actuated Transverse Displacement

**Figure 9:**
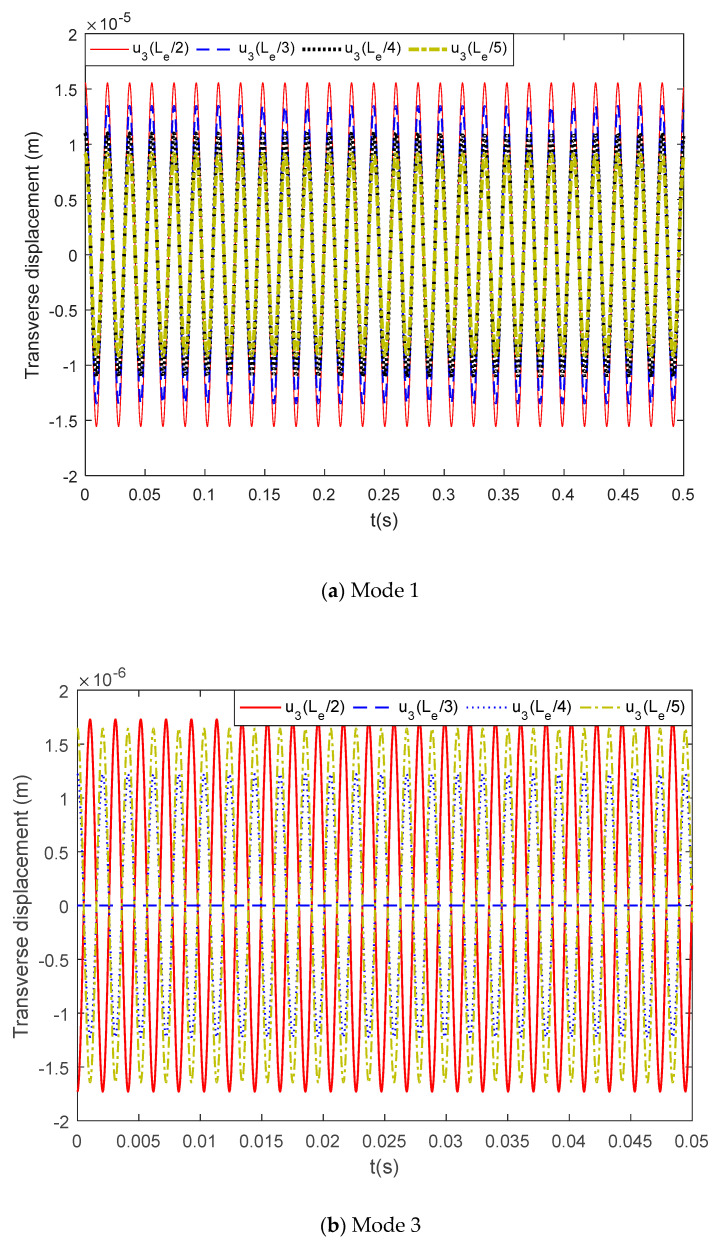
The flexoelectric induced transverse displacement, (**a**) mode 1, (**b**) mode 3.

The process of active vibration control usually is the cancellation of the initial vibration by the induced vibration. The flexoelectric actuator can induce a control force and moment in the structure and actuate dynamic responses correspondingly. The mode 1 and mode 3 controllable transverse displacement of the simply supported beam with flexoelectric actuating is plotted in Figure 9a,b, respectively.

It was found that for flexoelectric actuating, the controllable transverse displacement was in micron scale with an electric input amplitude of 1 V. For the mode 1 vibration, the transverse displacements $u_{3}\left(L_{e}/2\right)$, $u_{3}\left(L_{e}/3\right)$, $u_{3}\left(L_{e}/4\right)$, and $u_{3}\left(L_{e}/5\right)$ decreased constantly and were affected by the mode shape function. For the mode 3 vibration, the transverse displacement reached zero when $x=L_{e}/3$. The results were consistent with the influence of the probe location on the modal force and indicate that structure actuating and vibration control must be designed with consideration to the structure modes to achieve optimal effects.

## 4. Conclusions

With the flexoelectric effect, a simply supported beam was actuated. It proved that with a specific electric field gradient, the flexoelectric effect was still effective in macro-scale. In the presented work, an AFM probe and an electrode layer were utilized to generate an electric field gradient. Parameters that could affect the electric field gradient were studied and it was found that decreasing the probe radius could enhance the electric field gradient. When the flexoelectric layer thickness was decreased, the minimum values of the electric field gradient increased but the maximum values stayed the same, which made the effect of layer thickness on the gradient limited. The beam actuating effect depended on both the flexoelectric control moment and the structure modes. To optimize the actuating effect, flexoelectric actuators should be located at mode-dependent locations. Considering the electric field gradient’s effects on the flexoelectric actuating, flexoelectric actuating and control can be applied to engineering structures and feedback control strategies will be developed in future studies.

## Figures and Tables

**Table 1:** Key parameters of the physical model.

Properties	Values
Beam length *L_e_*, (m)	0.100
Beam width *b_e_*, (m)	0.010
Beam thickness, *h_e_* (m)	0.001
Young’s modulus of elastic beam, *Y*_e_ (N/m^2^)	1.556 × 10^9^
Beam mass density, *ρ* (kg/m^3^)	1100
Poisson’s ratio, *μ*	0.3
Flexoelectric patches thickness, *h_f_* (m)	50
Flexoelectric patches length, *L_f_* (m)	0.10
Flexoelectric constant, *π*_12_ (μV/m)	100
AFM probe tip radius, *R* (nm)	50
Actuation voltage, ϕ (V)	1
